# Enteroviral 3C protease cleaves N4BP1 to impair the host inflammatory response

**DOI:** 10.1128/jvi.01758-24

**Published:** 2024-12-10

**Authors:** Dongjie Zhang, Yifan Xie, Jie Cao, Lisu Huang, Wenchun Fan

**Affiliations:** 1 Zhejiang Provincial Key Laboratory for Cancer Molecular Cell Biology, Life Sciences Institute, Zhejiang University 127388 , Hangzhou, Zhejiang, China; 2 Department of Infectious Diseases, the Children's Hospital, Zhejiang University School of Medicine, National Clinical Research Center for Child Health 666702 , Hangzhou, Zhejiang, China; University of Kentucky College of Medicine, Lexington, Kentucky, USA

**Keywords:** enteroviruses, 3C protease, cleavage, N4BP1, inflammatory response

## Abstract

Enteroviral 3C protease (3Cpro) is an essential enzyme for viral replication and is responsible for combating the host anti-viral immune response by targeting cellular proteins for cleavage. The identification and characterization of 3Cpro substrates will contribute to our understanding of viral pathogenesis. In this study, we performed a motif search for 3Cpro substrates in the human protein database using FIMO, which refers to a common cleavage sequence of 3Cpro. We identified and characterized NEDD4-binding protein 1 (N4BP1), a key negative regulator of the NF-κB pathway, as a novel 3Cpro substrate. N4BP1 is cleaved at residue Q816 by 3Cpro from several human enteroviruses, resulting in the loss of its ability to regulate tumor necrosis factor alpha-activated NF-κB signaling. In addition, we found that mouse N4BP1, which has a threonine at the P1′ site, is resistant to human enteroviral 3Cpro cleavage. However, rodent enteroviral 3Cpro derived from encephalomyocarditis virus (EMCV) can cleave both human and mouse N4BP1 at a species-specific site. By combining bioinformatic, biochemical, and cell biological approaches, we identified and characterized N4BP1 as a novel substrate of enteroviral 3Cpro. These findings provide valuable insights into the interplay between 3Cpro, its substrates, and viral pathogenesis.

**IMPORTANCE:**

Targeting cellular proteins for cleavage by enteroviral 3Cpro is a conserved strategy used by enteroviruses to promote viral replication. While the cleavage of certain host proteins by 3Cpro may not affect viral replication, it is strongly associated with the pathogenesis of viral infection. In this study, we identified and characterized N4BP1, which plays such a role, using a combination of bioinformatic, biochemical, and cell biological approaches. Our data show that multiple 3Cpros cleave N4BP1 at residue Q816 and that cleavage of endogenous N4BP1 can occur during viral infection. N4BP1 has no effect on coxsackievirus B3 replication, but 3Cpro-induced N4BP1 cleavage abolishes its regulatory function in NF-κB signaling. We also show that mouse N4bp1 resists human enteroviral 3Cpro cleavage. In contrast, rodent enteroviral EMCV 3Cpro can target human and mouse N4BP1 for cleavage at different residues, which indicates that future investigations are needed to elucidate the potential mechanisms involved.

## INTRODUCTION

Human enteroviruses, which belong to the family *Picornaviridae*, possess a positive-stranded RNA genome of approximately 7,500 nucleotides containing a single open reading frame (ORF) flanked by untranslated regions (5′ and 3′ UTRs). The enteroviral ORF encodes a large precursor polyprotein that is processed into 11 mature viral proteins by viral proteases 2A (2Apro) and 3C (enteroviral 3C protease [3Cpro]) and unknown cellular proteases (1, 2). Four major enterovirus (EV) species have been identified in humans, ranging from type A to type D (EV-A to EV-D), including enterovirus A71 (EVA71), coxsackievirus A16 (CVA16), coxsackievirus B3 (CVB3), echovirus 11, poliovirus (PV), and enterovirus D68 (EVD68). Enteroviruses are highly prevalent worldwide and cause a variety of human diseases (3–5), such as hand-foot-and-mouth disease in children caused by EV-A (6), myocarditis and liver damage caused by EV-B (7, 8), poliomyelitis caused by EV-C (9), and acute flaccid myelitis caused by EVD68 (10, 11).

Viruses are obligate intracellular parasites that hijack and subvert the host cell machinery to create favorable conditions for viral replication. In the interplay between viruses and hosts, certain viral proteins play vital roles in viral replication, the suppression of host anti-viral innate immunity, and pathogenesis. Enteroviral 3Cpro is one such protein, responsible for the majority of the cleavage of the viral precursor polyprotein by recognizing the preferred consensus sequence of AXXQ/GPXX (X denotes any amino acid) and cleaving between glutamine and glycine residues (P1-Q and P1′-G site) (12). Using a similar mechanism, 3Cpro targets host proteins for cleavage, resulting in the suppression of host innate immune responses. For example, 3Cpro cleaves pattern recognition receptors (RIG-I and MAD5), key regulatory molecules (MAVS, TRIF, IRF7/9, and STAT1), and other host restriction factors (TRIM7 and OAS3) to evade host anti-viral strategies (13–19). Additionally, 3Cpro-mediated cleavage of host proteins is associated with viral pathogenesis; for instance, 3Cpro cleaves NLRP1 and CARD8, resulting in proinflammatory cell death (20, 21). Therefore, the broad identification and characterization of 3Cpro substrates can provide insights into virus‒host interactions, viral pathogenesis, and potential therapeutic targets for treating viral diseases. Multiple approaches have been used to identify 3Cpro substrates, such as bioinformatic analysis, two-dimensional gel electrophoresis, and proteomics-based terminal amine isotopic labeling of substrates (22–26).

In this study, we aimed to identify novel 3Cpro substrates that act as host decoys to activate host inflammatory responses and play important roles in viral pathogenesis but do not affect viral replication. Genes with such functions may be masked in functional screens *in vitro* due to the lack of suitable readout methods. Here, we used a bioinformatic approach to profile potential enteroviral 3Cpro substrates, predicting 7,395 potential substrates in the human proteome. One of the potential targets, NEDD4-binding protein 1 (N4BP1), attracted our interest because of its important role in the NF-κB signaling pathway. N4BP1 is a negative regulator of NF-κB signaling through inhibition of the recruitment of the kinase complex IKK to initiate the NF-κB pathway (27–29). N4BP1 is itself regulated by the activation of caspase 8, which cleaves N4BP1 in the domain linear ubiquitin-interacting (LUBIN) domain in N4BP1, which is a dimerization-dependent ubiquitin-binding module (at several sites: D311, D484, and D488) (30). The oligomerization of N4BP1 through the RNase domain generates a dimer that can bind M1-linked ubiquitin chains. This process is believed to allow the binding of NF-κB signaling essential modulator (NEMO), a component of the IKK complex, via its CoZi domain through ubiquitin linkages, resulting in the inhibition of the NF-κB pathway (31). However, N4BP1-mediated suppression of NF-κB could be inactivated by caspase 8 cleavage of N4BP1 at residues D424 and D490 (27). Our biochemical assays showed that human N4BP1 is targeted by 3Cpro for cleavage at glutamine 816 (Q816), which impairs its regulatory function in tumor necrosis factor alpha (TNFα)-triggered NF-κB-related inflammatory signaling but does not affect viral infection in tissue culture. In addition, we found that mouse N4bp1 is resistant to 3Cpro-mediated cleavage because of the threonine at its P1′ site. Taken together, our findings support the hypothesis that certain host proteins act as decoys for 3Cpro, which can trigger host inflammatory responses without compromising viral infection control (32).

## RESULTS

### Motif search for potential 3Cpro substrate profiles

**Fig 1:**
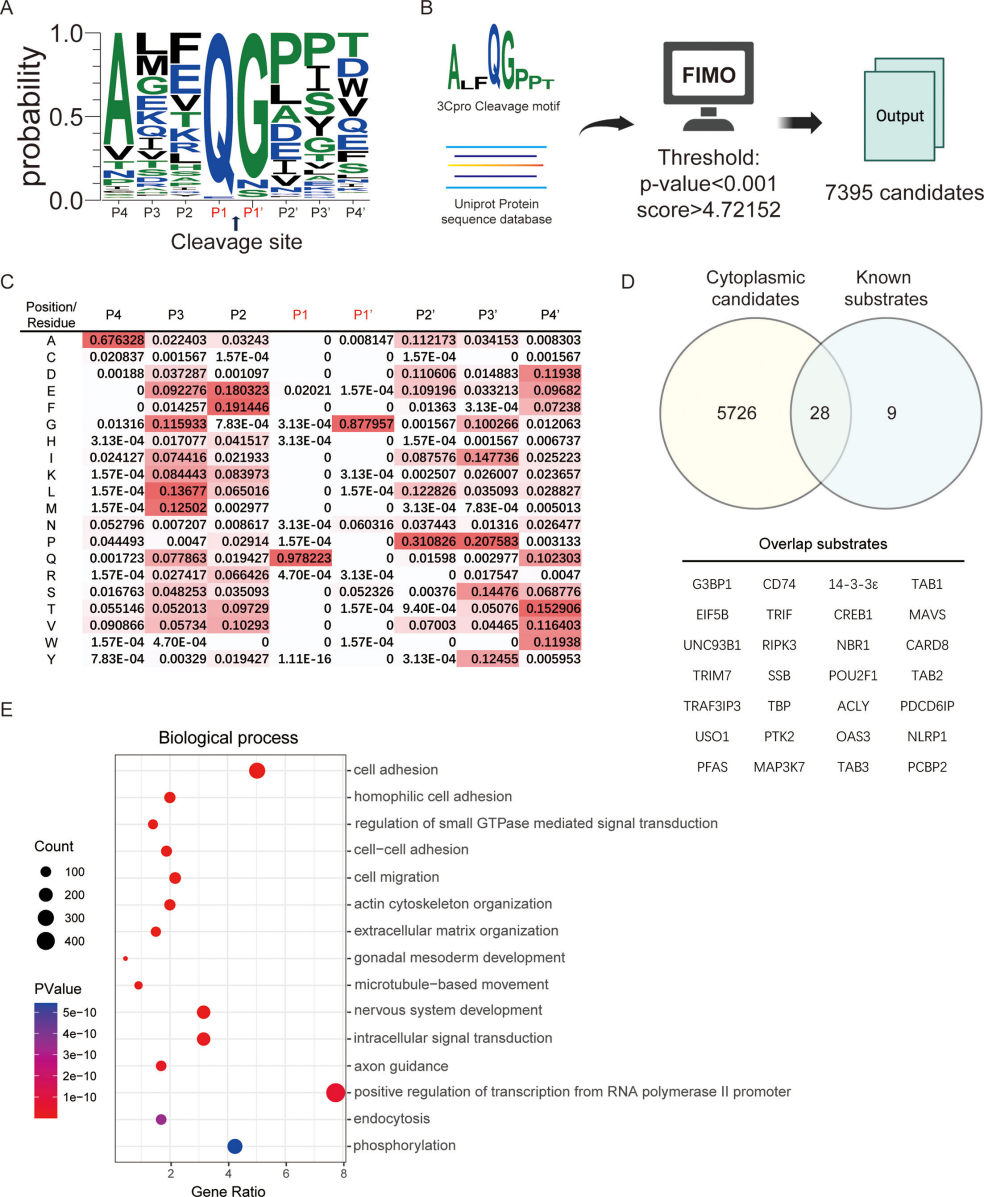
Motif search for potential enteroviral 3C protease (3Cpro) substrate profiles. (**A**) Eight-amino acid polyprotein cleavage motif (P4–P4′) of the enteroviral 3C protease. (**B**) Workflow for the search for potential substrates of the enteroviral 3C protease in the human protein database using FIMO. (**C**) Position probability matrix of the cleavage motif created by TBtools. (**D**) Venn diagram of the analysis of overlap between cytoplasmic candidate substrate proteins and experimentally validated proteins. The known substrate list is shown in Table S1. (**E**) The top 15 significant enriched Gene Ontology biological processes of cytoplasmic candidate substrate proteins.

We performed a motif search for enteroviral 3Cpro cleavage sites in human protein database using Find Individual Motif Occurrences (FIMO) (33) to identify potential host substrates of enteroviral 3Cpro. Tsu et al. referenced the enterovirus polyprotein cleavage sequences from the Virus Pathogen Resource (ViPR) (26) to generate the specific recognition motif of enteroviral 3Cpro. In addition, we incorporated experimentally validated substrate cleavage sequences. Moreover, instead of using the MEME Suite, we employed Weblogo3 and TBtools to generate the recognition motif and position probability matrix (PPM). For threshold adjustments, we utilized a comprehensive data set of human proteomic sequences rather than the enterovirus polyprotein data set or the reported human cleavage sites from a previous report (**34**). Finally, we generated an enteroviral 3Cpro cleavage motif, including the sequences containing four residues upstream and four residues downstream of the cleavage site (P4–P4′). We used the PPM of the cleavage motif with FIMO to search for and score human protein sequences in the UniProt protein database (35). We set the *P* value threshold to 0.001 to ensure that at least 75% of the experimentally verified substrate proteins were identified. Under these conditions, we identified 7,395 candidate substrate proteins containing potential 3Cpro cleavage sites (Fig. 1B and C). Given that 3Cpro functions in the cytoplasm following enterovirus infection, we further refined our candidate list by filtering for proteins localized to the cytoplasm, yielding 5,754 candidate substrate proteins, including 28 experimentally verified substrate proteins (Fig. 1D). Additionally, the Gene Ontology (GO) biological process enrichment analysis revealed that these candidate substrate proteins are predominantly involved in the regulation of transcription, cell adhesion, phosphorylation, and signal transduction, suggesting that 3Cpro may target proteins integral to these crucial cellular functions and potentially disrupt these processes during viral infection (Fig. 1E)**.**

### N4BP1 is cleaved by 3Cpro derived from multiple enteroviruses in a protease activity-dependent manner

**Fig 2:**
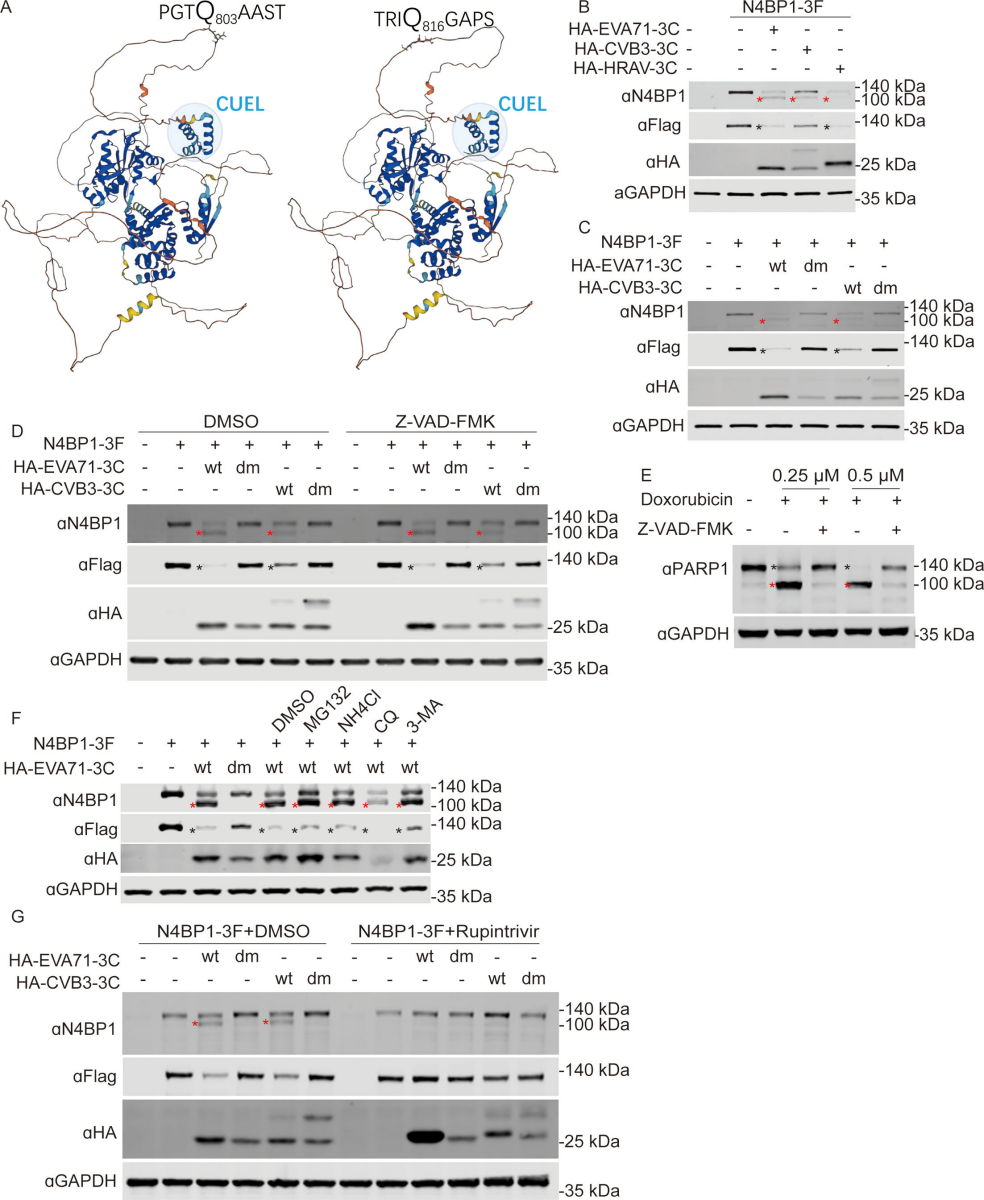
N4BP1 is cleaved by 3C proteases derived from multiple enteroviruses. (**A**) The structure of N4BP1 predicted by AlphaFold (40), and the predicted 3Cpro cleavage sites are labeled. (**B**) Western blot analysis of 293T cells co-transfected with plasmids expressing N4BP1-3F or HA-tagged 3Cpro derived from EVA71, CVB3, or HRVA. (**C**) Western blot analysis of lysates from 293T cells transfected with plasmids expressing N4BP1-3F and HA-tagged wild-type (wt) and catalytically dead 3Cpro (dm) derived from EVA71 and CVB3. (**D**) Western blot analysis of lysates from 293T cells transfected with plasmids expressing N4BP1-3F and HA-tagged wild-type or catalytically dead 3Cpro derived from EVA71 and CVB3 with or without Z-VAD-FMK treatment (10 µM). (**E**) HeLa cells were treated with doxorubicin at a final concentration of 0.25 or 0.5 µM with or without Z-VAD-FMK for 24 h. The cell lysates were subjected to Western blot analysis with the indicated antibodies. (**F**) Western blot analysis of 293T cells co-transfected with plasmids expressing N4BP1-3F and HA-tagged 3Cwt or 3Cdm of EVA71 with or without treatment with the indicated reagents. (**G**) Western blot analysis of lysates from 293T cells transfected with plasmids expressing N4BP1-3F and HA-tagged 3Cwt or 3Cdm derived from EVA71 and CVB3 with or without treatment with the panenteroviral 3C inhibitor rupintrivir (1 µM). The Western blots are representative of three independent experiments showing similar results. (B–D and F) Red asterisks denote cleaved N4BP1, and black asterisks denote full-length N4BP1. (E) Red asterisks denote cleaved PARP1, and dark asterisks denote full-length PARP1. (B–D, F, and G) The cells were harvested at 24 h post-transfection.

A plasmid expressing C-terminal 3Flag-tagged N4BP1 (N4BP1-3F) was co-transfected with an N-terminal HA-tagged 3Cpro-expressing plasmid (HA-3C) into 293T cells, followed by Western blot analysis to assess whether human N4BP1 can be targeted for cleavage by enteroviral 3Cpro. As shown in Fig. 2A, in the presence of 3Cpro expression derived from EVA71, CVB3, and human rhinovirus A (HRVA), a cleavage product of N4BP1 was detected using an antibody against N4BP1. In addition, the level of the full-length N4BP1-3F protein was reduced in the presence of the indicated 3Cpro, as shown by 3Flag-tag antibody staining. Next, we investigated whether the protease activity is required for 3Cpro-mediated N4BP1 cleavage. For this experiment, we co-transfected plasmids expressing N4BP1-3F and HA-tagged wild-type (wt) or protease catalytically deficient (H40A and C147A) 3Cpro (3Cdm) from EVA71 and CVB3 into 293T cells. The results revealed that the N4BP1 cleavage product was observed only in the presence of catalytically active 3Cpro but not 3Cdm (Fig. 2B and C), indicating that the catalytic activity of the protease is required for 3Cpro to cleave N4BP1. We investigated whether enteroviral 3Cpro-mediated cleavage of N4BP1 is associated with the cellular processes induced by 3Cpro, such as 3Cpro-induced activation of caspases (36). Therefore, we evaluated 3Cpro-mediated N4BP1 cleavage in the presence of the apoptosis inhibitor Z-VAD-FMK. We found that Z-VAD-FMK did not abolish 3Cpro-mediated cleavage of N4BP1 but inhibited doxorubicin-triggered cleavage of PARP1 by caspase (Fig. 2D and E). Furthermore, other inhibitors, such as the 26S proteasome inhibitor MG132, the lysosome inhibitor NH4Cl, and the autophagy inhibitors chloroquine (CQ) and 3-methyladenine (3-MA), failed to block the 3Cpro-mediated cleavage of N4BP1 (Fig. 2F). Furthermore, rupintrivir, an enteroviral 3Cpro inhibitor (37–39), was also included to assess its role in 3Cpro-induced N4BP1 cleavage. We found that addition of rupintrivir abolished 3Cpro-mediated cleavage of N4BP1 (Fig. 2G). Taken together, these results demonstrate that human N4BP1 is a novel substrate of 3Cpro from several enteroviruses, including EVA71, CVB3, and HRVA, and that the catalytic activity of 3Cpro is essential for the 3Cpro-mediated cleavage of N4BP1.

### Enteroviral 3Cpro cleaves N4BP1 at glutamine 816

**Fig 3:**
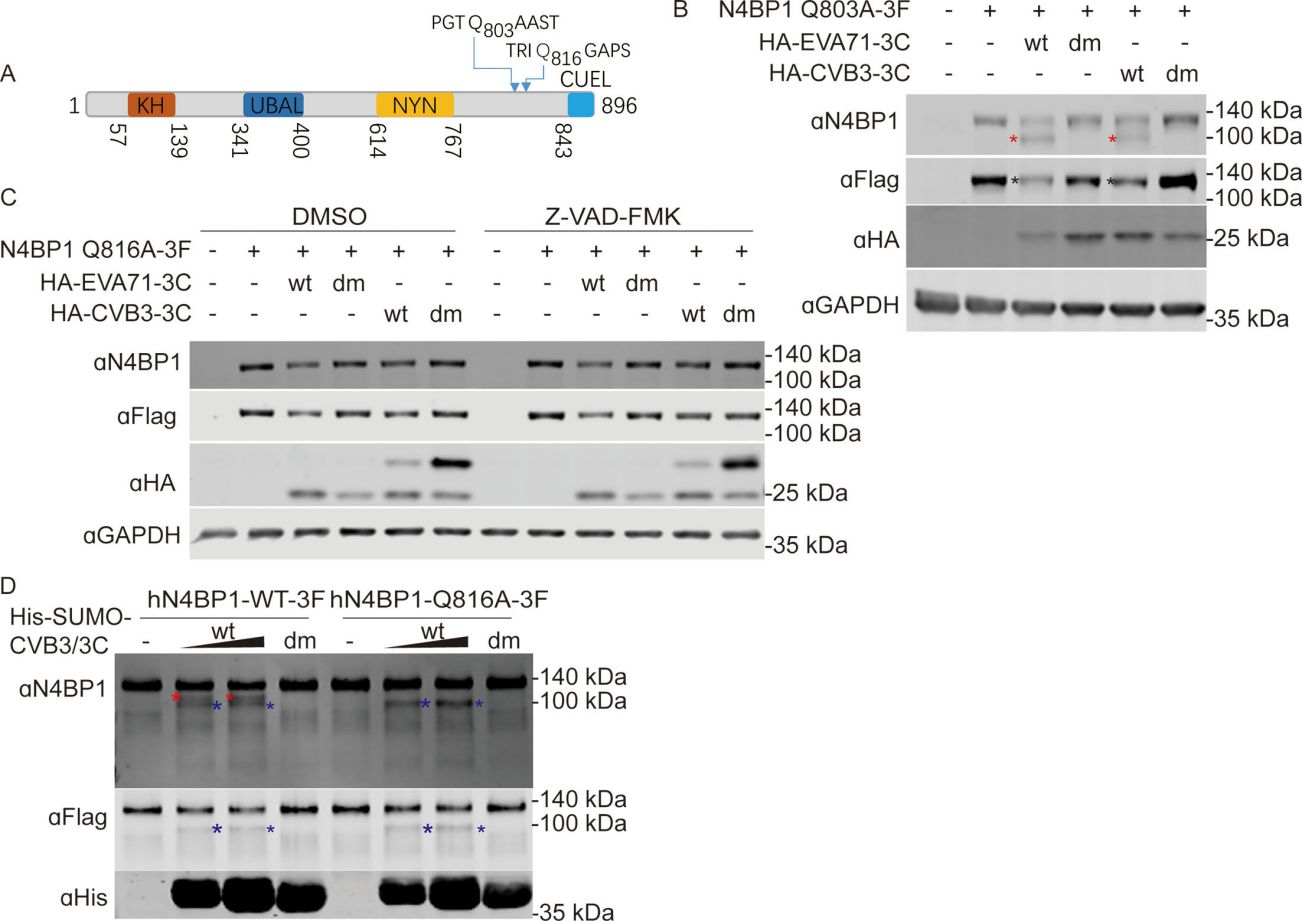
Enteroviral 3C protease cleaves N4BP1 at glutamine 816. (**A**) Schematic illustration of the human N4BP1 protein and the predicted 3Cpro cleavage sites. (**B**) Western blot analysis of 293T cells co-transfected with plasmids expressing N4BP1Q803A-3F and HA-tagged 3Cwt or 3Cdm derived from EVA71 or CVB3. (**C**) Western blot analysis of lysates from 293T cells transfected with plasmids expressing N4BP1Q816A-3F and HA-tagged 3Cwt or 3Cdm derived from EVA71 and CVB3 with or without Z-VAD-FMK treatment. (**D**) *In vitro* cleavage assay of N4BP1. Anti-Flag resin-purified hN4BP1WT-3F (5 µg) and hN4BP1Q816A-3F (5 µg) were incubated with purified His-SUMO-3Cwt (5 and 10 µg) or His-SUMO-3Cdm (10 µg) at the indicated concentrations at 37°C for 2 h, followed by Western blotting. The Western blots are representative of three independent experiments showing similar results. Red and blue asterisks denote cleaved N4BP1, and dark asterisks denote full-length N4BP1.

Two potential cleavage sites, Q803 and Q816, located in a disordered region of the C-terminus of N4BP1 were identified (Fig. 3A). We mutated Q803 and Q816 of N4BP1 to alanine (N4BP1–Q803A and N4BP1–Q816A) to test whether Q803 or Q816 is the cleavage site. Two mutant N4BP1-expressing plasmids were subsequently co-transfected with HA-3Cwt or HA-3Cdm into 293T cells, and N4BP1 cleavage was assessed via Western blotting. Our results showed that the N4BP1-Q803A mutant was still cleaved by 3Cwt from EVA71 and CVB3 (Fig. 3B). However, the N4BP1–Q816A mutant resisted 3Cpro-mediated cleavage (Fig. 3C). Furthermore, we also performed an *in vitro* cleavage assay to confirm whether N4BP1 can be cleaved directly by CVB3 3Cpro. For this goal, CVB3 3Cwt and 3Cdm were expressed and purified from *Escherichia coli*. N4BP1WT-3F and N4BP1Q816A-3F were purified from HeLa cells stably expressing N4BP1WT-3F and N4BP1Q816A-3F using anti-Flag beads. The *in vitro* cleavage assay was subsequently conducted, as previously described (41). As shown in Fig. 3D, two cleaved bands with very similar molecular weights were observed for N4BP1WT-3F in the presence of 3Cwt but not 3Cdm. Interestingly, the lower-molecular-weight band of N4BP1Q816A-3F remained after the incubation with 3Cwt. Based on the molecular weights of cleaved fragments, we speculated that the cleaved fragment with the larger molecular weight may be produced by 3C cleavage at Q816 in N4BP1. The other fragment may be cut at the Q803 residue in N4BP1. Taken together, our data indicate that human N4BP1 is a novel substrate of enteroviral 3Cpro and is cleaved at glutamine 816.

### CVB3 infection results in the cleavage of endogenous N4BP1

**Fig 4:**
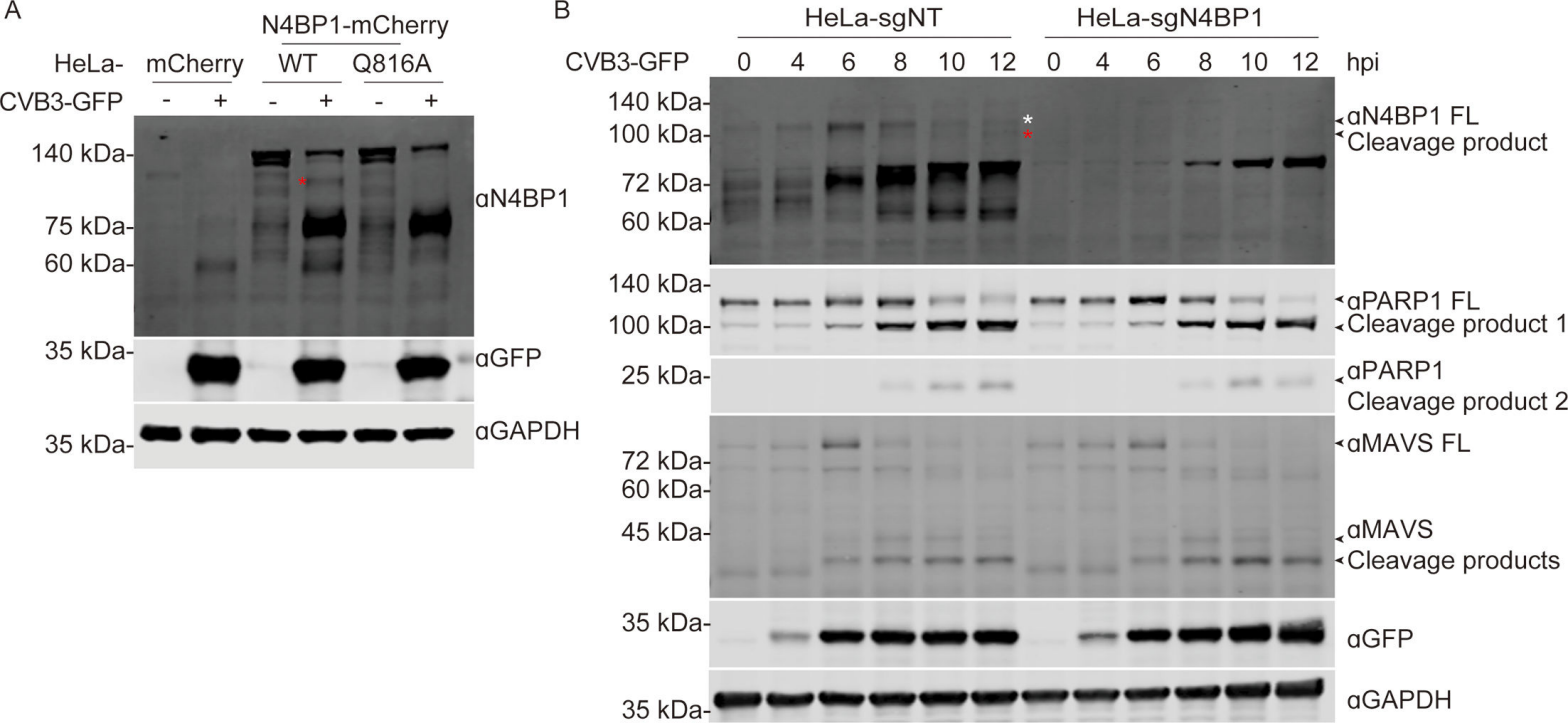
Endogenous N4BP1 undergoes cleavage during CVB3 infection. (**A**) HeLa cells expressing mCherry, N4BP1WT-mCherry, or N4BP1Q816A-mCherry were infected with CVB3–GFP at an MOI of 5 for 12 h. Then, Western blotting was conducted on the cell lysates using the indicated antibodies. (**B**) HeLa-sgNT or HeLa-sgN4BP1 cells were infected with CVB3–GFP at an MOI of 5 for the indicated times. The cleavage of endogenous N4BP1, MAVS, and PARP1 was analyzed by Western blot analysis using the indicated antibodies. The Western blots are representative of three independent experiments showing similar results. Red asterisks denote cleaved N4BP1, and white asterisks denote full-length N4BP1.

We aimed to confirm that 3Cpro-mediated cleavage of N4BP1 occurs in the context of viral infection. We first established HeLa cells with stable expression of N4BP1-3F, N4BP1-WT-mCherry, N4BP1Q816A-mCherry, mCherry, or the empty vector. The cells were infected with CVB3–GFP at a multiplicity of infection (MOI) of 5, and N4BP1 cleavage was assessed by Western blotting. As shown in Fig. 4A, a specific cleavage product was observed only for N4BP1WT-mCherry but not N4BP1Q816A-mCherry in the presence of CVB3–GFP infection. Additionally, we also observed a reduction in the level of the full-length N4BP1Q816A protein during viral infection, which may be related to the inhibition of translation by viral infection. Finally, we generated N4BP1-deficient HeLa cells to assess N4BP1 cleavage induced by CVB3 infection. As shown in Fig. 4B, a reduction in the level of N4BP1 and the cleavage product was observed in HeLa-sgNT cells during CVB3–GFP infection. Moreover, two known host substrates of CVB3, PAPR1 and MAVS, exhibited similar cleavage dynamics in both HeLa sgNT and HeLa N4BP1 knockout cells. Taken together, these results show that human N4BP1 is capable of being cleaved during CVB3 infection.

### N4BP1 has no significant role in CVB3 infection in tissue cultures

**Fig 5:**
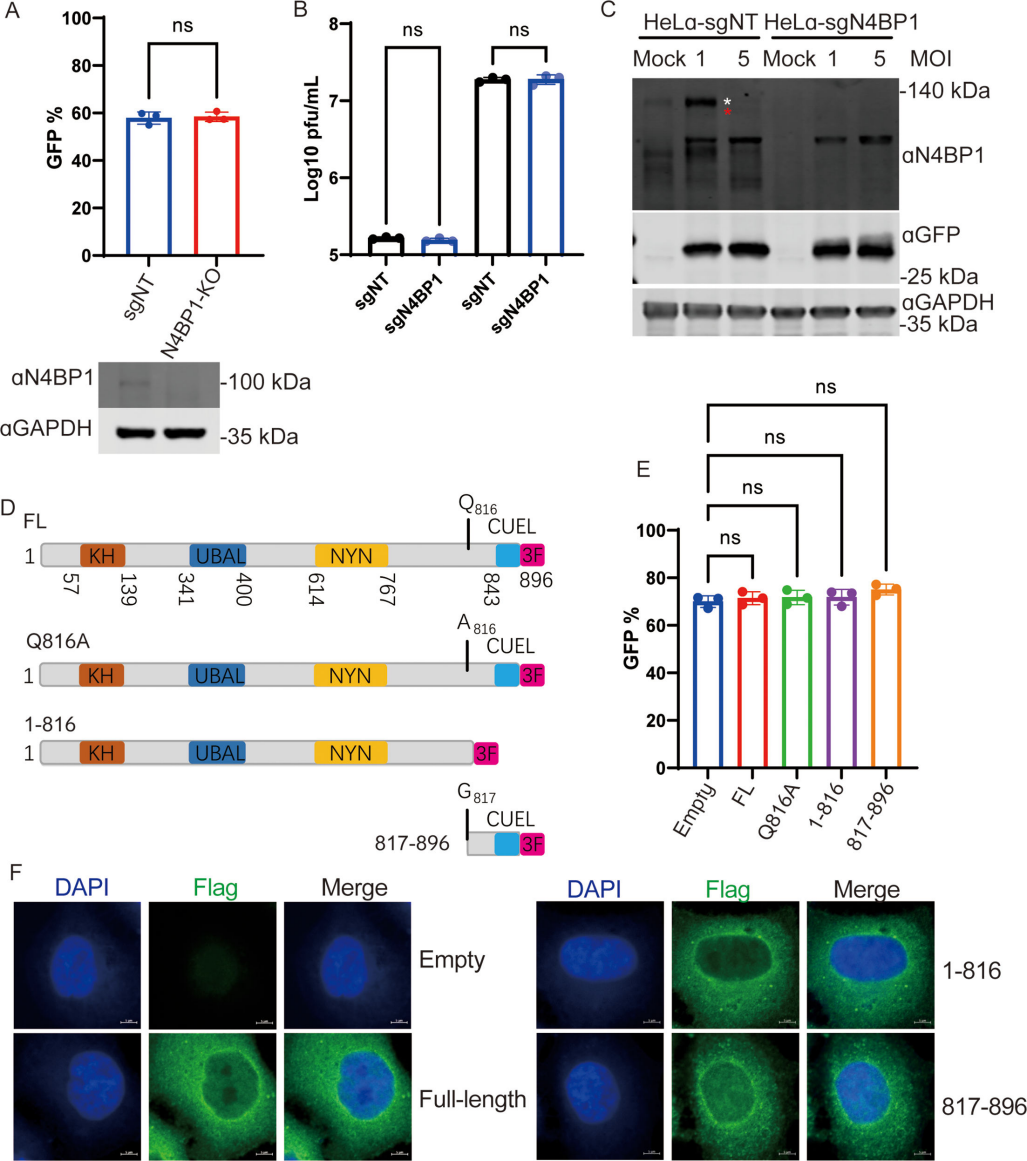
N4BP1 has no obvious effect on CVB3 replication. (**A**) HeLa-sgNT and HeLa-sgN4BP1 cells were infected with CVB3–GFP at an MOI of 0.01 for 24 h. The degree of virus infectivity was determined by flow cytometry (*n* = 3). (Bottom panel) N4BP1 expression detected using Western blot assays. (**B**) HeLa-sgNT and HeLa-sgN4BP1 cells were infected with CVB3–GFP at an MOI of 1 or 5 for 12 h. Virus production was determined by plaque assay (*n* = 3). (**C**) Cell lysates from panel **B** were analyzed by Western blotting using the indicated antibodies. (**D**) Schematic illustration of full-length and truncated N4BP1, which refers to enteroviral 3Cpro cleavage products. (**E**) HeLa cells expressing the indicated N4BP1 constructs were infected with CVB3–GFP at an MOI of 0.01 for 24 h. The degree of virus infectivity was determined by flow cytometry (*n* = 3). (**F**) HeLa cells were transfected with plasmids expressing 3× Flag-tagged full-length N4BP1 and 3Cpro-cleaved N4BP1. The intracellular distribution of N4BP1 was analyzed using an immunofluorescence assay. Anti-Flag staining, green; 4′,6-diamidino-2-phenylindole, blue. Scale bar, 5 µm. In panels A, B, and E, the data represent the averages of independent biological replicates (*n* = 3) and are presented as means ± SDs. Student’s unpaired *t*-test was used (ns, *P* > 0.05). ns, not significant.

We investigated the effect of N4BP1 on CVB3 infection. To this end, we assessed the viral infectivity of CVB3–GFP in N4BP1-deficient HeLa cells generated via CRISPR-Cas9. These results indicated that the loss of N4BP1 had no obvious effect on CVB3 infection (Fig. 5A through C). Next, we investigated the effects of the products of N4BP1 cleaved by 3Cpro on CVB3 infection. We generated HeLa cells with stable expression of full-length, 3Cpro-resistant mutant and truncated N4BP1 according to the 3Cpro cleavage site (Fig. 5D). As shown in Fig. 5E, compared with that in empty control cells, the infectivity of CVB3 in HeLa cells stably expressing the indicated N4BP1 constructs was barely affected. Finally, the intracellular distributions of the full-length and cleaved N4BP1 fragments were examined. As shown in Fig. 5F, no significant changes in the cleavage products of N4BP1 were observed compared with its full-length version. Taken together, these data suggest that the expression of N4BP1 and 3Cpro-cleaved N4BP1 has no effect on CVB3 infection in tissue culture.

### 3Cpro-mediated N4BP1 cleavage products fail to inhibit TNFα-triggered NF-κB signaling

**Fig 6:**
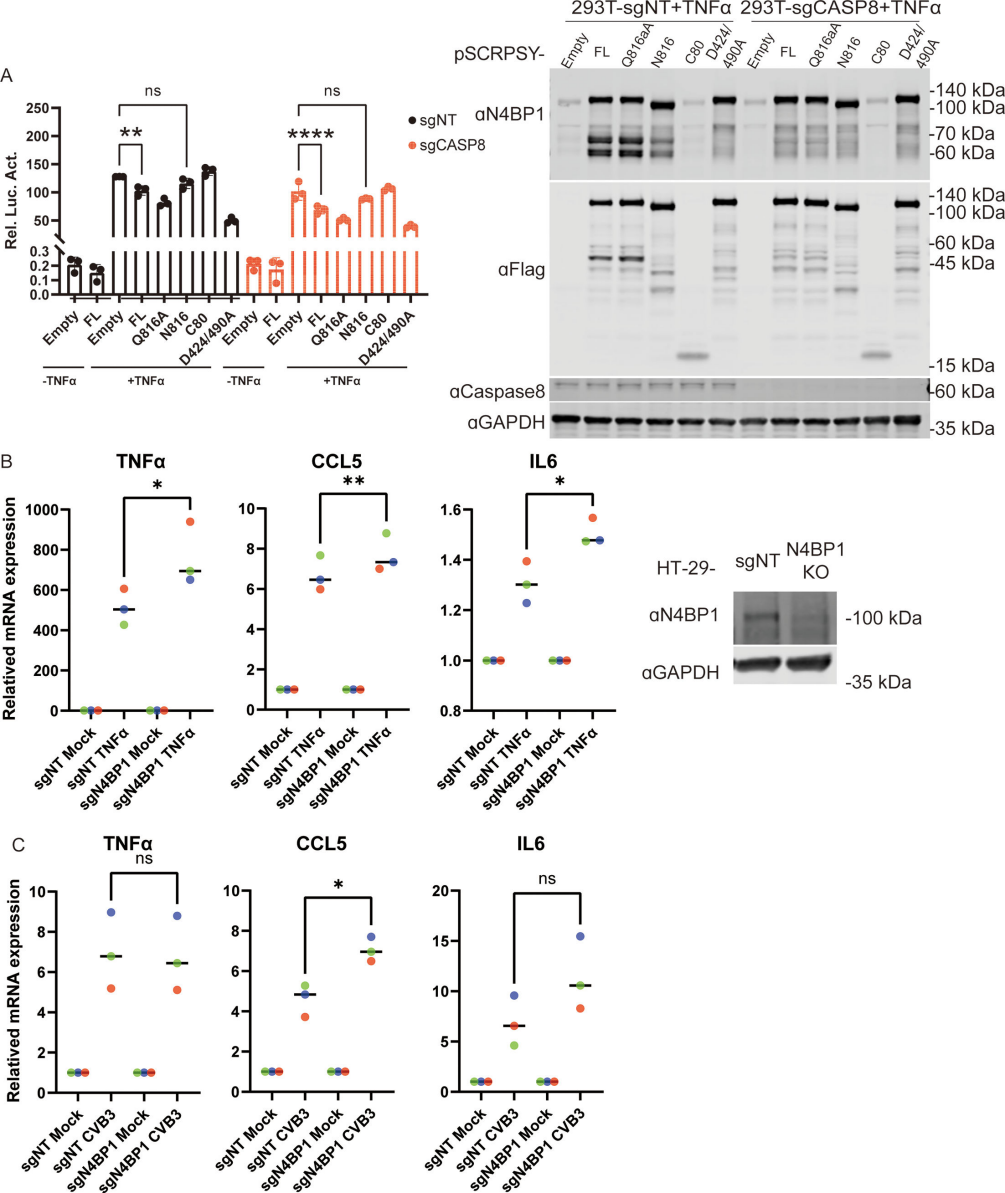
The molecular function of N4BP1 is altered after cleavage by 3Cpro. (**A**) (Left panel) NF-κB-dependent luciferase reporter activity in 293T-sgNT or 293T-sgCASP8 cells transfected with the empty vector or vectors encoding full-length N4BP1 and its mutants and stimulated with TNFα (+TNFα, 20 ng/mL) or unstimulated (−TNFα) for 6 h. Ordinary one-way analysis of variance was used (ns, *P* > 0.05; **P < 0.01, ****P < 0.0001). Right panel, immunoblot analysis of 3× Flag-tagged N4BP1 or truncation mutants and caspase 8 in the analyzed cells. (**B**) (Left panel) Reverse transcription‒quantitative PCR (RT‒qPCR) analysis of the mRNA levels of TNFα, CCL5, and IL6 in wild-type or N4BP1^−/−^ HT-29 cells treated with TNFα (60 ng/mL) for 12 h. (Right panel) Immunoblot analysis of endogenous N4BP1 levels in wild-type or N4BP1^−/−^ HT-29 cells. (**C**) RT‒qPCR was used to analyze the mRNA levels of TNFα, CCL5, and IL6 in wild-type or N4BP1^−/−^ HT-29 cells infected with CVB3–GFP at an MOI of 1 for 12 h. (B and C) The mRNA levels were normalized to the GAPDH mRNA levels, and the mRNA levels in the treatment group were normalized to those in the mock group. Student’s paired *t*-test (two-tailed) was used (ns, *P* > 0.05; *P < 0.05; **P < 0.01). All the data represent the averages of independent biological replicates (*n* = 3) and are presented as means ± SDs. ns, not significant.

Since N4BP1 negatively regulates the NF-κB signaling pathway, we proceeded to assess the effects of 3Cpro-mediated N4BP1 cleavage fragments on TNFα-activated NF-κB signaling. 293T-sgNT or 293T-sgCASP8 cells were transiently transfected with the NF-κB luciferase reporter plasmid together with various N4BP1 expression plasmids or the empty control plasmid. As an internal control, pRL-TK was transfected simultaneously. Twenty-four hours post-transfection, the cells were stimulated with TNFα for 6 h. Compared with full-length N4BP1, the 3Cpro-cleaved fragments 1–816 and 817–896 were not functional in NF-κB signaling (Fig. 6A). In contrast, the N4BP1–Q816A mutant retained its inhibitory function. Consistent with previous reports, the N4BP1 D424/490A mutant, which resists caspase 8-mediated cleavage, had a stronger inhibitory effect on TNFα-induced NF-κB activation than the WT and other N4BP1 mutants in 293T-sgNT cells. Similar results were observed in caspase-8-deficient cells. We also established N4BP1 knockout HT-29 cells and investigated the regulatory effects of N4BP1 on TNFα and CVB3 infection-induced NF-κB signaling. Consistent with previous studies, the loss of N4BP1 increased the expression levels of inflammatory cytokines, including TNFα, CCL5, and IL6 (Fig. 6B). Next, we found that CVB3 infection upregulated the expression of TNFα, CCL5, and IL6 (Fig. 6C). However, the level of CCL5 was only slightly upregulated in N4BP1 knockout cells compared with wild-type cells in the context of CVB3 infection (Fig. 6C). These data suggest that 3Cpro-mediated cleavage of the N4BP1 may abolish N4BP1-mediated inhibition of the inflammatory response.

### Mouse N4BP1 resists human enteroviral 3Cpro cleavage because it contains a threonine residue at the P1′ position

**Fig 7:**
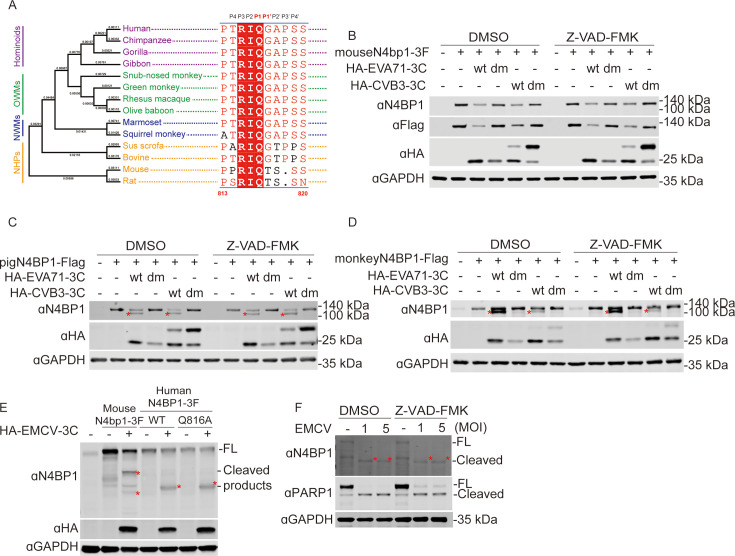
Mouse N4bp1 resists enteroviral 3Cpro-mediated cleavage. (**A**) Phylogenetic analysis of N4BP1 and amino acid alignment of the enteroviral 3Cpro cleavage sequence. (**B–D**) Western blot analysis of 293T cells co-transfected with plasmids expressing N4BP1 derived from mouse (mouse N4BP1-3F, **B**), pig (pig N4BP1-F, **C**), and monkey (monkey N4bp1-F, **D**) cell lines and HA-tagged 3Cwt or 3Cdm derived from EVA71 or CVB3, respectively. The cells were harvested at 24 h post-transfection for Western blot analysis. (**E**) Western blot analysis of lysates from 293T cells transfected with plasmids expressing mouse N4bp1-3F, human N4BP1WT-3F, and human N4BP1Q816A-3F and HA-tagged 3C derived from EMCV. (**F**) HeLa cells were infected with EMCV at MOIs of 1 and 5 for 24 h. The cleavage of endogenous N4BP1 and PARP1 was analyzed by Western blot analysis using the indicated antibodies. The Western blots are representative of three independent experiments showing similar results. Red asterisks denote cleaved N4BP1.

We aligned N4BP1 proteins from 14 mammalian species to investigate the diversity of N4BP1 throughout evolution. We found that the 3Cpro cleavage motif in human and nonhuman primate N4BP1 is identical and highly conserved in pig and bovine orthologs. Notably, the mouse and rat orthologs contain a threonine (T) residue at the P1′ position, which is inconsistent with the enteroviral 3Cpro cleavage motif (Fig. 7A and Fig. 1A). Therefore, we speculated that mouse N4bp1 might resist enteroviral 3Cpro-mediated cleavage. We validated this hypothesis by performing an *in vivo* cleavage assay using mouse N4bp1 in 293T cells co-transfected with wild-type or catalytically mutated 3Cpro from EVA71 and CVB3 for 24 h, followed by Western blot analysis. Indeed, we found that 3Cwt derived from EVA71 and CVB3 failed to cleave mouse N4bp1 (Fig. 7B). In addition, we observed a significant reduction in the level of full-length N4bp1 in the presence of 3Cwt compared with 3Cdm, and the caspase inhibitor Z-VAD-FMK was unable to reverse this reduction (Fig. 7B). We also examined the cleavage of monkey and porcine N4BP1, which contain the conserved 3Cpro cleavage motif. As shown in Fig. 7C and D, both pig and monkey N4BP1 retained sensitivity to wild-type enteroviral 3Cpro. We also found that the caspase inhibitor Z-VAD-FMK was unable to abolish 3Cpro-mediated cleavage. EVA71 and CVB3 are human enteroviruses that do not circulate in rodents. Therefore, we asked whether the rodent enteroviral 3Cpro can cleave mouse N4BP1. Next, we evaluated the cleavage potential of 3Crpo derived from encephalomyocarditis virus (EMCV) in mouse N4bp1 and human N4BP1WT and N4BP1Q816A. Interestingly, we found that all the tested N4BP1 protein could be cleaved by EMCV 3Cpro to produce species-specific cleavage fragments (Fig. 7E). Notably, a similar cleavage product was observed for WT and Q816A N4BP1, which is different from human enteroviral 3Cpro-mediated cleavage. This finding indicates that EMCV recognizes a different cleavage site in human N4BP1. Furthermore, a similar cleaved fragment of N4BP1 was also observed in HeLa cells infected with EMCV, and the apoptosis inhibitor Z-VAD-FMK did not abolish the EMCV-induced cleavage of N4BP1 (Fig. 7F). Taken together, our data highlight the evolutionary conflicts between enteroviruses and their hosts.

## DISCUSSION

Enteroviral 3C protease is a multifunctional enzyme that plays critical roles in viral replication and in the regulation of host inflammatory responses. Through its proteolytic activities, 3Cpro modulates several signaling pathways, including those involved in the immune response, apoptosis and cellular stress responses (13, 42, 43). Thus, 3Cpro not only facilitates viral replication but also contributes to the pathological consequences of enterovirus infection. The identification and characterization of the bona fide host substrates of 3Cpro could lead to a better understanding of the mechanisms of interaction between enteroviruses and the host, provide valuable insights into viral pathogenesis, and may aid in the development of targeted anti-viral therapies.

Using N-terminomics approaches, the Jagdeo et al. and Saeed et al. defined the proteolytic landscape during enterovirus infection and identified hundreds of novel 3Cpro substrates (23, 24). In the work of Jagdeo et al. and Saeed et al., the cleavage of 3Cpro substrates was well characterized. However, the biological effects of 3Cpro-mediated substrate cleavage remain to be elucidated. Knockdown, knockout, and overexpression are the most commonly used strategies to assess the effects of 3Cpro substrates on viral replication. However, substrates that do not produce a phenotype are often easily overlooked because of the lack of an ideal test system and scenario. Therefore, identifying 3Cpro substrates and elucidating their biological significance have been a long-standing challenge. We applied a bioinformatic approach that uses the PPM of the cleavage motif of 3Cpro to search for and score human protein sequences in the UniProt protein database as a method to expand the potential 3Cpro substrates. Under conditions where the *P* value was less than 0.001 and the score was greater than 4.72152, 7,395 candidates were predicted (Fig. 1). Among thousands of potential substrates, we focused on N4BP1 in the present study. N4BP1 is a multifunctional protein with diverse roles in regulating ubiquitination, signal transduction, protein‒protein interactions, and immune responses (36–39, 41, 42, 44, 45), but its role in enterovirus infection is poorly understood. Two cleavage sites were predicted in the C-terminus of N4BP1, namely, Q803 and Q816, but only the Q816 site was targeted for 3Cpro cleavage (Fig. 2). Notably, Q803 is the first predicted cleavage site of 3Cpro in N4BP1, but the Q803A mutant is still cleaved by 3Cpro (Fig. 3B). We then explored another potential cleavage site around Q803 that could produce similar cleavage fragments. Finally, Q816 was identified as the target for 3Cpro (Fig. 3C) by resetting the search parameters.

N4BP1 contains four functional domains, from the N-terminus to the C-terminus: the KH, UBAL, NYN, and CUEL (known as CoCUN) domains. 3Cpro-mediated cleavage of N4BP1 isolates the CUEL domain from the KH–UBAL–NYN motif (Fig. 3). The N4BP1 CUEL domain is a ubiquitin-binding domain (46) that is critical for its regulatory function in NF-κB signaling by modulating N4BP1 dimerization (29, 30). Thus, we hypothesized that 3Cpro targets N4BP1 for cleavage to relive N4BP1-mediated inhibition of NF-κB signaling, which may enhance the inflammatory response caused by viral infection. Indeed, 3Cpro-induced cleavage of N4BP1 fragments failed to suppress TNFα-triggered NF-κB signaling (Fig. 6A). Consistent with previous studies, TNFα induced the production of higher levels of inflammatory cytokines in N4BP1-deficient cells than in wild-type cells (Fig. 6B). However, we observed only a modest increase in the inflammatory response in N4BP1-deficient cells infected with CVB3 compared with that in uninfected cells (Fig. 6C). One possible explanation is that certain viral proteins also play a role in inhibiting the inflammatory response in our tissue culture cell model. For example, CVB3 3Cpro can also cleave the NF-κB cytosolic inhibitor IκBα to produce a fragment that retains NF-κB inhibition in the nucleus (47). Oberst et al. reported that N4BP1 inhibits the E3 ligase ITCH by binding to ITCH through its CUEL domain, resulting in the inhibition of ITCH autoubiquitylation and ITCH-mediated ubiquitylation of p73α (48). In addition, the CUEL domain of N4BP1 is also responsible for the N4BP1-mediated degradation of the notch intracellular domain to promote neural stem cell differentiation (49). Therefore, we speculate that 3Cpro-mediated cleavage of N4BP1 plays a role in modulating its molecular function.

To date, controversy persists over whether enteroviruses can be transmitted across species. However, a systematic review of the literature concerning enteroviruses suggested that enteroviruses may be zoonotic, particularly among nonhuman primates (50). In this study, we found that the human enteroviruses EVA71, CVB3, and HRAV 3Cpro can target human, monkey, and pig N4BP1, but not mouse N4bp1, for cleavage at a specific residue. In contrast, the rodent enterovirus EMCV 3Cpro can cleave mouse and human N4BP1 at different residues. These findings suggest that an evolutionary conflict exists between enteroviral 3Cpro and their natural hosts. However, the subsequent effect of 3Cpro-mediated cleavage of N4BP1 is poorly understood. One possibility is that N4BP1 acts as an effector for sensing enteroviral 3Cpro to trigger the host immunity response (32). In conclusion, we identified and validated N4BP1 as a novel substrate of enteroviral 3Cpro that is specifically cleaved at glutamine 816. While the cleavage of N4BP1 does not significantly affect CVB3 infection *in vitro*, it impairs its regulatory function in NF-κB signaling. Furthermore, the cleavage resistance observed in mouse N4bp1 suggests an evolutionary mechanism to evade viral proteolysis. Therefore, our findings expand our understanding of host–virus interactions and reveal a novel molecular mechanism by which 3Cpro regulates the host inflammatory response. However, our study has several limitations, and further studies are needed to elucidate the biological significance of enteroviral 3Cpro-mediated N4BP1 cleavage:

1. Mouse N4bp1 resists 3Cpro-mediated cleavage, but the level of the full-length protein still decreases in the presence of 3Cpro, highlighting the need for additional studies to understand the interplay between 3Cpro and mouse N4bp1.
2. We did not observe an increased inflammatory response in N4BP1 knockout cells compared with wild-type cells in the presence of CVB3 infection, and additional primary cells or an *in vivo* model would be helpful to refine the biological significance. We could not clarify the subsequent effects of enteroviral 3Cpro-mediated cleavage of N4BP1 at this moment. However, the physiological and direct cleavage of N4BP1 by enteroviral 3Cpro suggests that N4BP1 may be an effector that senses enteroviral proteases to trigger the host response (32).
3. In addition to the regulation of NF-κB signaling, further studies are needed to determine the effects of 3Cpro-induced cleavage of N4BP1 fragments in the context of enterovirus infection.

## MATERIALS AND METHODS

### MOTIF search

Polyprotein sequences of enteroviruses were collected from the ViPR database (51). From these sequences, the 3Cpro cleavage sites were extracted, specifically including the sequences four residues upstream and four residues downstream of the cleavage site (P4–P4′), following the methodology described by Tsu et al. (26). A total of 37 experimentally validated 3Cpro substrate protein cleavage sites (P4–P4′) in humans were compiled from the literature. Using these sequences, a specific motif for the 3Cpro cleavage sites was generated with WebLogo3 (52) and used to create a PPM with TBtools (53). Human proteome sequences were downloaded from the UniProt Swiss-Prot protein database and searched using FIMO against the 3Cpro cleavage site-specific motif with a *P* value threshold of 0.001. This analysis identified 7,395 candidate substrate proteins. GO (54, 55) annotations were then used to filter for proteins localized to the cytoplasm, refining the list to 5,754 candidate proteins, including 28 known substrate proteins.

### Cell cultures

293T and HeLa cells were maintained in Dulbecco’s modified Eagle medium (DMEM) (Gibco) supplemented with 10% fetal bovine serum (FBS, xCell), 1% penicillin–streptomycin (Yeason), and 1× nonessential amino acids (NEAAs, Biosharp). HT-29 cells were maintained in RPMI-1640 (Procell) supplemented with 10% FBS (ExCell), 1% penicillin–streptomycin (Yeason), and 1× NEAAs (Biosharp). All cells were cultured at 37°C with 5% CO_2_.

### Virus and viral infection

The propagation of virus and infection of cells with CVB3–GFP and VEEV–GFP was conducted as previously described (56, 57). The virus infectivity of CVB3–GFP in cells was analyzed using flow cytometry and plaque assay. EMCV strain BJC3 (GenBank accession no. DQ464062) was kindly provided by Dr. Shu Zhu (Zhejiang University) (58).

### Antibodies and reagents

Antibodies used included mouse anti-Flag (Proteintech, catalog no. 66008-4-Ig), rabbit anti-HA tag (Proteintech, catalog no. 51064-2-AP), rabbit anti-N4BP1 (Abclonal, catalog no. A8474N), rabbit anti-GFP tag (Proteintech, catalog no. 50430-2-AP), mouse anti-PARP1 (Proteintech, catalog no. 66520-1-Ig), rabbit anti-MAVs (Abclonal, catalog no.14341–1-AP), rabbit anti-Caspase8 (Proteintech, catalog no. 13423-1-AP), and mouse anti-GAPDH (ABclonal, catalog no. AC033). Dimethyl sulfoxide (catalog no. 67-68-5) was purchased from Yeason. TNFα (catalog no. 300-01A) was purchased from Peprotech. CQ (catalog no. HY-17589A), proteasome inhibitor MG132 (catalog no. 133407-82-6), and pan-caspase inhibitor Z-VAD-FMK (catalog no. 161401-82-7) were purchased from MedChemExpress. Pan-enteroviral 3C inhibitor rupintrivir (catalog no. 223537-30-2) was purchased from GLPBIO. 3-MA (M9281) was purchased from Sigma-Aldrich.

### Plasmids and reporters

The C-terminal 3× Flag-tagged wild-type N4BP1 and its mutants Q803A and Q816A and mouse N4bp1 were cloned into the lentiviral vector pLVX.EnCMV.IRES.mCherry. The C-terminal Flag-tagged monkey N4BP1 and pig N4BP1 were kindly provided by Dr. Fang He (Zhejiang University) (59). The C-terminal 3× Flag-tagged wild-type N4BP1 and its mutants **Δ**N816, ΔC80, and D424/490A and the C-terminal mCherry-tagged wild-type N4BP1 and its mutant Q816A were moved into the lentiviral vector pSCRPSY-DEST by Gateway cloning, using pENTR plasmids in an LR reaction (Invitrogen). Enteroviral (EVA71, CVB3, and HRVA) and EMCV 3C-expressing constructs were N-terminally HA tagged based on the pTT-HA expression vector (60). The individual 3C coding sequences were referred to Enterovirus A71 strain TW-00073–2012 (GenBank accession no. MG756724) (61), Coxsackievirus B3 Woodruff variant (GenBank accession no. U57056) (62), human rhinovirus A strain HRV-A01_p1103_sR1167_2009 (GenBank accession no. JN815255) (63), and EMCV strain BJC3 (GenBack accession no. DQ464062) (58), respectively. The H40A/C147A double catalyzed active site mutants EVA71 and CVB3 3C (3Cdm) were generated by overlap extension using the polymerase chain reaction as previously described (64).

NF-κB luciferase reporter plasmid and control plasmid pRL-TK were kindly provided by Dr. Yongqun Zhu (Life Sciences Institute, Zhejiang University) (65). Wild-type or CASP8 knockout 293T cells seeded on 24-well plates were transiently transfected with 50 ng of the luciferase reporter plasmid together with a total of 300 ng of various expression plasmids or empty control plasmid. As an internal control, 10 ng of pRL-TK was transfected simultaneously. Twenty-four hours after transfection, the cells were stimulated with TNFα (20 ng/mL) for 6 h. Dual luciferase activity in the total cell lysates was quantified using Dualucif Firefly and Renilla Assay Kit (UElandy, catalog no. F6075).

### Lentivirus production and transduction

293T cells (7 × 10^5^) in six-well plates were co-transfected with plasmids expressing wild-type N4BP1 or mutants, HIV-1 gag–pol, and VSV-G in a ratio of 1.0:0.8:0.2, respectively. Five microliters polyethyleneimine HCl MAX (Polysciences) was combined with 2 µg total DNA in 100 µL Opti-MEM (Gibco). Transfections were carried out for 6 h, followed by a medium change to DMEM containing 3% FBS. Supernatants were collected at 48 and 72 h, pooled, cleared by centrifugation, and stored at −80°C. For lentiviral transduction, HeLa cells were seeded into 12-well plates at a density of 5 × 10^4^ cells per well and transduced with lentivirus by spin inoculation at 1,000 × *g* at 37°C for 45 min in a medium containing 3% FBS, 20 mM HEPES, and 4 µg/mL polybrene.

### *In vivo* cleavage assay

293T cells were co-transfected wild-type or mutated N4BP1−3× Flag-expressing constructs (200 ng) with HA-tagged 3Cwt- or 3Cdm-expressing plasmid (800 ng) in a 24-well plate. The cells were harvested in NP-40 lysis buffer (100 mM Tris–HCl [pH 7.5], 100 mM NaCl, 10 mM EDTA, and 1% NP-40 in sterilized water), followed by Western blot analysis of the N4BP1 cleavage. To examine the cleavage of N4BP1 in the context of virus infection, HeLa cells stably expressing N4BP1WT-3Flag, N4BP1Q816A-3F Flag, N4BP1D424/490 A-3F Flag, N4BP1WT-mCherry, and N4BP1Q816A-mCherry were infected with CVB3–GFP for 6 h. To examine the cleavage of endogenous N4BP1 during virus infection, HeLa-sgNT cells and HeLa-sgN4BP1 cells were infected with CVB3–GFP for 4, 6, 8, 10, and 12 h.

### *In vitro* cleavage assay

3Flag-tagged N4BP1–WT and N4BP1–Q816A were purified from HeLa cells stably expressing N4BP1–WT-3F and N4BP1–Q816A–3F using anti-Flag resin (Selleck, catalog no. B26102) and eluted by Flag–peptide (Genscript, catalog no. RP10586CN). The elution buffer was prepared by diluting Flag-peptide in buffer containing 100 mM Tris–HCl (pH 7.5), 150 mM NaCl, and 1 mM dithiothreitol (DTT) at a final concentration of 200 ng/mL. His-SUMO-tagged wild-type and catalytically inactive CVB3 3C proteases were expressed and purified from *E. coli*. The *in vitro* cleavage assay of N4BP1 was performed by incubating N4BP1–WT–3F (5 µg) and N4BP1–Q816A–3F (5 µg) with His-SUMO-3Cwt (5 and 10 µg) or His-SUMO-3Cdm (10 µg) in a 25-µL reaction containing 50 mM HEPES (pH 7.5), 3 mM EDTA, 150 mM NaCl, 0.005% (vol/vol) Tween-20, and 10 mM DTT at 37°C for 2 h, followed by Western blot analysis of the N4BP1 cleavage.

### Western blot and co-immunoprecipitation analysis

Tissue culture cells were lysed in Nonidet P-40 (NP-40) buffer (50 mM Tris [pH 7.4], 150 mM NaCl, 1% [vol/vol] NP-40, 1 mM EDTA, protease inhibitor cocktail) and incubated on ice for 10 min, followed by centrifugation at 13,000 rpm for 10 min at 4°C. For co-immunoprecipitation assays, plasmids expressing genes of interest were co-transfected into 293T cells in a six-well plate. The cells were harvested at 30 h post-transfection and lysed in 400 µL NP-40 lysis buffer containing protease inhibitors. After incubation on ice for 10 min, lysates were cleared by centrifugation at 13,000 rpm for 10 min at 4°C. Subsequently, a portion of the cell lysate was saved for analysis as input, and another portion was subjected to precipitation with anti-Flag magnetic beads (Selleck, catalog no. B26102) overnight at 4°C with rocking. The next day, the beads or gel were washed seven times with immunoprecipitation wash buffer (50 mM Tris [pH 7.4], 500 mM NaCl, 0.1% [vol/vol] NP-40, and 1 mM EDTA). Precipitated proteins were eluted from beads or gel by heating sample in SDS loading buffer at 95°C for 10 min.

### Immunofluorescence analysis and confocal microscopy

Cells were fixed using 4% paraformaldehyde, permeabilized with 0.1% saponin in 1× phosphate-buffered saline (PBS) containing 3% bovine serum albumin (BSA) at 4°C for 20 min, and blocked in 1× PBS containing 3% BSA at room temperature (RT) for 1 h. Then, the cells were incubated with the indicated primary antibodies in 1× PBS containing 1% BSA for 1 h at RT. Cells were washed five times with PBS and incubated with secondary antibodies in 1× PBS containing 1% BSA for 1 h at room temperature in the dark, followed by five washes with PBS and staining with ProLong Diamond anti-fade mountant with 4′,6-diamidino-2-phenylindole (Sigma, catalog no. D9542).

For confocal immunofluorescence studies, the indicated antibodies and dyes were diluted as follows: mouse anti-Flag (Proteintech, catalog no.66008-4-Ig), 1:1,000, and goat anti-mouse Ig–Alexa Fluor 488 (Invitrogen), 1:1,000.

### CRISPR-Cas9-mediated gene editing

CRISPR gRNA sequences were chosen from Human CRISPR Knockout Pooled Library (Brunello) (66), and corresponding oligos were purchased from Tsingke Biotech. The oligos were cloned into the Cas9-expressing lentiCRISPRv2 plasmid (a gift from F. Zhang, Addgene plasmid 52961) as previously described (67). 293T cells in six-well plates were co-transfected with the cloning products, HIV-1 gag–pol, and VSV-G in a ratio of 1.0:0.7:0.5, respectively. HeLa, 293T, and HT-29 cells were transduced with the collected lentivirus. At 48 h post-transduction, the cells were screened by puromycin or blasticidin for 2 weeks and then verified by Western blot. CRISPR gRNA sequences used included sgNT (5′-AGTAGACGGACGGTGAGCTG-3), sgN4BP1 (5′-AGAAAGAGAATGTTACCCCA-3′), and sgCASP8 (5′-GCCTGGACTACATTCCGCAA-3′).

### Quantitative real-time PCR analysis

Cells were seeded on 12-well plates and incubated overnight before being infected by CVB3–GFP with the indicated MOI. At designated time points, cell lysates were harvested using the RNAeasy Viral RNA Isolation Kit with Spin Column (Beyotime, catalog no. R0035L) according to the manufacturer’s protocol. After the reverse transcription reaction using HiScript III 1st Strand cDNA Synthesis Kit (Vazyme, catalog no. R312-02), quantitative PCR (qPCR) was performed using ChamQ Universal SYBR qPCR Master Mix (Vazyme, catalog no. Q311-03). RNA levels were normalized to GAPDH RNA levels.

The following primers (5′–3′ orientation) were used for the qPCR: hIL6-Fwd: AGACAGCCACTCACCTCTTCAG, hIL6-Rev: TTCTGCCAGTGCCTCTTTGCTG; hIL1β-Fwd: AAATACCTGTGGCCTTGGGC, hIL1β-Rev: TTTGGGATCTACACTCTCCAGCT; hCCL5-Fwd: GGCAGCCCTCGCTGTCATCC, hCCL5-Rev: GCAGCAGGGTGTGGTGTCCG; hTNFα-Fwd: GCCGCATCGCCGTCTCCTAC, hTNFα-Rev: CCTCAGCCCCCTCTGGGGTC; and hGAPDH-Fwd: GGTGGTCTCCTCTGACTTCAACA, hGAPDH-Rev: GTTGCTGTAGCCAAATTCGTTGT.

### Statistics

All data are presented as means and standard deviations. Data were analyzed using GraphPad Prism software (version 9.0.0). Individual statistical tests are specified in the figure legends. In general, at least three independent biological replicates were carried out for each experiment. Data were reproduced in independent experiments, as indicated in the figure legends.

## Data Availability

All relevant data supporting the findings of this study are included within the article and supplemental material.

## SUPPLEMENTAL MATERIAL

Table S1 jvi.01758-24-s0001.docx Known substrates of enteroviral 3CPro.

ASM does not own the copyrights to Supplemental Material that may be linked to, or accessed through, an article. The authors have granted ASM a non-exclusive, world-wide license to publish the Supplemental Material files. Please contact the corresponding author directly for reuse.
