# Supplementary material for: Enteroviral 3C protease cleaves N4BP1 to impair the host inflammatory response
Source: J Virol. 2024 Dec 10;99(1):e01758-24. doi: 10.1128/jvi.01758-24 (PMC11784292; doi:10.1128/jvi.01758-24)
Supplement: Table S1 — Known substrates of enteroviral 3CPro. [file jvi.01758-24-s0001.docx]

Table S1 Known substrates of enteroviral 3CPro

| **Known substrates** | **Uniprot Accession number** | **Cleavage site** | **Reference(PMID)** |
| --- | --- | --- | --- |
| ZAP(ZC3HAV1) | Q7Z2W4 | TNDQ⬇GARR | 29182509 |
| UNC93B1 | Q9H1C4 | AGPQ⬇GDED | 26509685 |
| TRIM7 | Q9C029 | AELQ⬇GEAT | 36106874 |
| TRIF(TICAM1) | Q8IUC6 | AFPQ⬇SLPF | 21436888 |
| TRIF(TICAM1) | Q8IUC6 | AGPQ⬇SLPL | 21436888 |
| TRAF3IP3 | Q9Y228 | AREQ⬇GPSR | 35814660 |
| TDP-43(TARDBP) | Q13148 | AAAQ⬇AALQ | 37039659 |
| TBP | P20226 | ASPQ⬇GAMT | 8388502 |
| TAB3 | Q8N5C8 | SAMQ⬇GPSP | 24942571 |
| TAB3 | Q8N5C8 | YQKQ⬇GSHS | 24942571 |
| TAB2 | Q9NYJ8 | GQLQ⬇GGQS | 24942571 |
| TAB1 | Q15750 | THTQ⬇SSSS | 24942571 |
| TAB1 | Q15750 | MPSQ⬇GQMV | 24942571 |
| SSB | P05455 | VQFQ⬇GKKT | 9971802 |
| RIPK3 | Q9Y572 | AERQ⬇GMNW | 29449668 |
| RELA(p65) | Q04206 | LLNQ⬇GIPV | 15845545 |
| PTK2 | Q05397 | GSLQ⬇GPIG | 22341464 |
| POU2F1(OCT1) | P14859 | GFTQ⬇GDVG | 9426457 |
| PFAS | O15067 | VQVQ⬇GDNT | 29437971 |
| PCBP2 | Q15366 | AMQQ⬇SHFP | 24371074 |
| PABPC1 | P11940 | VHVQ⬇GQEP | 11836384 |
| PABPC1 | P11940 | WTAQ⬇GARP | 11836384 |
| **Known substrates** | **Uniprot Accession number** | **Cleavage site** | **Reference(PMID)** |
| PABPC1 | P11940 | AIPQ⬇TQNR | 11836384 |
| P115(USO1) | O60763 | VEVQ⬇GETE | 29437971 |
| OAS3 | Q9Y6K5 | AWEQ⬇GGKD | 35504537 |
| NLRP1 | Q9C000 | GCTQ⬇GSER | 33093214 |
| NFKBIA(IκBα) | P25963 | VTYQ⬇GYSP | 17351338 |
| NBR1 | Q14596 | ALPE⬇GPLG | 24769734 |
| MAVS | Q7Z434 | QETQ⬇APES | 21436888 |
| MAP3K7(TAK1) | O43318 | AKQQ⬇SESG | 28424289;24942571 |
| IRF7 | Q92985 | AVQQ⬇SCLA | 26608321;23175366 |
| HNRNPM | P52272 | IAKQ⬇GGGG | 29437971 |
| hNRNPK | P61978 | YEPQ⬇GGSG | 29437971 |
| G3BP1 | Q13283 | AGEQ⬇GDIE | 30006004;24260247 |
| EIF5B | O60841 | VMEQ⬇GVPE | 18572216 |
| CSTF2 | P33240 | ASMQ⬇GGVP | 19779565 |
| CREB1 | P16220 | AITQ⬇GGAI | 8995645 |
| CD74 | P04233 | ALPQ⬇GPMQ | 37409968 |
| CARD8 | Q9Y2G2 | IRLQ⬇GSRK | 37289745 |
| ALIX(PDCD6IP) | Q8WUM4 | PAYG⬇SSPA | 29437971 |
| ACLY | P53396 | AKNQ⬇ALKE | 29437971 |
| 14-3-3ε(YWHAE) | P62258 | SDMQ⬇GDGE | 37555661 |

**Refrences for table 1:**

1. Xie L, Lu B, Zheng Z, Miao Y, Liu Y, Zhang Y, Zheng C, Ke X, Hu Q, Wang H. The 3C protease of enterovirus A71 counteracts the activity of host zinc-finger antiviral protein (ZAP). J Gen Virol. 2018 Jan;99(1):73-85. doi: 10.1099/jgv.0.000982. Epub 2017 Nov 28. PMID: 29182509.
2. Harris KG, Coyne CB. Unc93b Induces Apoptotic Cell Death and Is Cleaved by Host and Enteroviral Proteases. PLoS One. 2015 Oct 28;10(10):e0141383. doi: 10.1371/journal.pone.0141383. PMID: 26509685.
3. Fan W, McDougal MB, Schoggins JW. Enterovirus 3C Protease Cleaves TRIM7 To Dampen Its Antiviral Activity. J Virol. 2022 Oct 12;96(19):e0133222. doi: 10.1128/jvi.01332-22. Epub 2022 Sep 15. PMID: 36106874.
4. Mukherjee A, Morosky SA, Delorme-Axford E, Dybdahl-Sissoko N, Oberste MS, Wang T, Coyne CB. The coxsackievirus B 3C protease cleaves MAVS and TRIF to attenuate host type I interferon and apoptotic signaling. PLoS Pathog. 2011 Mar;7(3):e1001311. doi: 10.1371/journal.ppat.1001311. Epub 2011 Mar 10. PMID: 21436888.
5. Li H, Yao Y, Chen Y, Zhang S, Deng Z, Qiao W, Tan J. TRAF3IP3 Is Cleaved by EV71 3C Protease and Exhibits Antiviral Activity. Front Microbiol. 2022 Jun 23;13:914971. doi: 10.3389/fmicb.2022.914971. PMID: 35814660.
6. Zhang L, Yang J, Li H, Zhang Z, Ji Z, Zhao L, Wei W. Enterovirus D68 Infection Induces TDP-43 Cleavage, Aggregation, and Neurotoxicity. J Virol. 2023 Apr 27;97(4):e0042523. doi: 10.1128/jvi.00425-23. Epub 2023 Apr 11. PMID: 37039659.
7. Das S, Dasgupta A. Identification of the cleavage site and determinants required for poliovirus 3CPro-catalyzed cleavage of human TATA-binding transcription factor TBP. J Virol. 1993 Jun;67(6):3326-31. doi: 10.1128/JVI.67.6.3326-3331.1993. PMID: 8388502.
8. Lei X, Han N, Xiao X, Jin Q, He B, Wang J. Enterovirus 71 3C inhibits cytokine expression through cleavage of the TAK1/TAB1/TAB2/TAB3 complex. J Virol. 2014 Sep 1;88(17):9830-41. doi: 10.1128/JVI.01425-14. Epub 2014 Jun 18. PMID: 24942571.
9. Shiroki K, Isoyama T, Kuge S, Ishii T, Ohmi S, Hata S, Suzuki K, Takasaki Y, Nomoto A. Intracellular redistribution of truncated La protein produced by poliovirus 3Cpro-mediated cleavage. J Virol. 1999 Mar;73(3):2193-200. doi: 10.1128/JVI.73.3.2193-2200.1999. PMID: 9971802.
10. Lötzerich M, Roulin PS, Boucke K, Witte R, Georgiev O, Greber UF. Rhinovirus 3C protease suppresses apoptosis and triggers caspase-independent cell death. Cell Death Dis. 2018 Feb 15;9(3):272. doi: 10.1038/s41419-018-0306-6. PMID: 29449668.
11. Neznanov N, Chumakov KM, Neznanova L, Almasan A, Banerjee AK, Gudkov AV. Proteolytic cleavage of the p65-RelA subunit of NF-kappaB during poliovirus infection. J Biol Chem. 2005 Jun 24;280(25):24153-8. doi: 10.1074/jbc.M502303200. Epub 2005 Apr 21. PMID: 15845545.
12. Bozym RA, Delorme-Axford E, Harris K, Morosky S, Ikizler M, Dermody TS, Sarkar SN, Coyne CB. Focal adhesion kinase is a component of antiviral RIG-I-like receptor signaling. Cell Host Microbe. 2012 Feb 16;11(2):153-66. doi: 10.1016/j.chom.2012.01.008. PMID: 22341464.
13. Yalamanchili P, Weidman K, Dasgupta A. Cleavage of transcriptional activator Oct-1 by poliovirus encoded protease 3Cpro. Virology. 1997 Dec 8;239(1):176-85. doi: 10.1006/viro.1997.8862. PMID: 9426457.
14. Jagdeo JM, Dufour A, Klein T, Solis N, Kleifeld O, Kizhakkedathu J, Luo H, Overall CM, Jan E. N-Terminomics TAILS Identifies Host Cell Substrates of Poliovirus and Coxsackievirus B3 3C Proteinases That Modulate Virus Infection. J Virol. 2018 Mar 28;92(8):e02211-17. doi: 10.1128/JVI.02211-17. PMID: 29437971.
15. Chase AJ, Daijogo S, Semler BL. Inhibition of poliovirus-induced cleavage of cellular protein PCBP2 reduces the levels of viral RNA replication. J Virol. 2014 Mar;88(6):3192-201. doi: 10.1128/JVI.02503-13. Epub 2013 Dec 26. PMID: 24371074.
16. Kuyumcu-Martinez NM, Joachims M, Lloyd RE. Efficient cleavage of ribosome-associated poly(A)-binding protein by enterovirus 3C protease. J Virol. 2002 Mar;76(5):2062-74. doi: 10.1128/jvi.76.5.2062-2074.2002. PMID: 11836384.
17. Zhou X, Tian L, Wang J, Zheng B, Zhang W. EV71 3C protease cleaves host anti-viral factor OAS3 and enhances virus replication. Virol Sin. 2022 Jun;37(3):418-426. doi: 10.1016/j.virs.2022.04.013. Epub 2022 May 3. PMID: 35504537.
18. Robinson KS, Teo DET, Tan KS, Toh GA, Ong HH, Lim CK, Lay K, Au BV, Lew TS, Chu JJH, Chow VTK, Wang Y, Zhong FL, Reversade B. Enteroviral 3C protease activates the human NLRP1 inflammasome in airway epithelia. Science. 2020 Dec 4;370(6521):eaay2002. doi: 10.1126/science.aay2002. Epub 2020 Oct 22. PMID: 33093214.
19. Saura M, Lizarbe TR, Rama-Pacheco C, Lowenstein CJ, Zaragoza C. Inhibitor of NF kappa B alpha is a host sensor of coxsackievirus infection. Cell Cycle. 2007 Mar 1;6(5):503-6. doi: 10.4161/cc.6.5.3918. Epub 2007 Mar 25. PMID: 17351338.
20. Shi J, Fung G, Piesik P, Zhang J, Luo H. Dominant-negative function of the C-terminal fragments of NBR1 and SQSTM1 generated during enteroviral infection. Cell Death Differ. 2014 Sep;21(9):1432-41. doi: 10.1038/cdd.2014.58. Epub 2014 Apr 25. PMID: 24769734.
21. Rui Y, Su J, Wang H, Chang J, Wang S, Zheng W, Cai Y, Wei W, Gordy JT, Markham R, Kong W, Zhang W, Yu XF. Disruption of MDA5-Mediated Innate Immune Responses by the 3C Proteins of Coxsackievirus A16, Coxsackievirus A6, and Enterovirus D68. J Virol. 2017 Jun 9;91(13):e00546-17. doi: 10.1128/JVI.00546-17. PMID: 28424289.
22. Xiang Z, Liu L, Lei X, Zhou Z, He B, Wang J. 3C Protease of Enterovirus D68 Inhibits Cellular Defense Mediated by Interferon Regulatory Factor 7. J Virol. 2015 Nov 25;90(3):1613-21. doi: 10.1128/JVI.02395-15. PMID: 26608321.
23. Lei X, Xiao X, Xue Q, Jin Q, He B, Wang J. Cleavage of interferon regulatory factor 7 by enterovirus 71 3C suppresses cellular responses. J Virol. 2013 Feb;87(3):1690-8. doi: 10.1128/JVI.01855-12. Epub 2012 Nov 21. PMID: 23175366.
24. Zhang Y, Yao L, Xu X, Han H, Li P, Zou D, Li X, Zheng L, Cheng L, Shen Y, Wang X, Wu X, Xu J, Song B, Xu S, Zhang H, Cao H. Enterovirus 71 inhibits cytoplasmic stress granule formation during the late stage of infection. Virus Res. 2018 Aug 15;255:55-67. doi: 10.1016/j.virusres.2018.07.006. Epub 2018 Jul 10. PMID: 30006004.
25. Fung G, Ng CS, Zhang J, Shi J, Wong J, Piesik P, Han L, Chu F, Jagdeo J, Jan E, Fujita T, Luo H. Production of a dominant-negative fragment due to G3BP1 cleavage contributes to the disruption of mitochondria-associated protective stress granules during CVB3 infection. PLoS One. 2013 Nov 18;8(11):e79546. doi: 10.1371/journal.pone.0079546. PMID: 24260247.
26. de Breyne S, Bonderoff JM, Chumakov KM, Lloyd RE, Hellen CU. Cleavage of eukaryotic initiation factor eIF5B by enterovirus 3C proteases. Virology. 2008 Aug 15;378(1):118-22. doi: 10.1016/j.virol.2008.05.019. Epub 2008 Jun 24. PMID: 18572216.
27. Weng KF, Li ML, Hung CT, Shih SR. Enterovirus 71 3C protease cleaves a novel target CstF-64 and inhibits cellular polyadenylation. PLoS Pathog. 2009 Sep;5(9):e1000593. doi: 10.1371/journal.ppat.1000593. Epub 2009 Sep 25. PMID: 19779565.
28. Yalamanchili P, Datta U, Dasgupta A. Inhibition of host cell transcription by poliovirus: cleavage of transcription factor CREB by poliovirus-encoded protease 3Cpro. J Virol. 1997 Feb;71(2):1220-6. doi: 10.1128/JVI.71.2.1220-1226.1997. PMID: 8995645.
29. Xiang Z, Tian Z, Wang G, Liu L, Li K, Wang W, Lei X, Ren L, Wang J. CD74 Interacts with Proteins of Enterovirus D68 To Inhibit Virus Replication. Microbiol Spectr. 2023 Aug 17;11(4):e0080123. doi: 10.1128/spectrum.00801-23. Epub 2023 Jul 6. PMID: 37409968.
30. Tsu BV, Agarwal R, Gokhale NS, Kulsuptrakul J, Ryan AP, Fay EJ, Castro LK, Beierschmitt C, Yap C, Turcotte EA, Delgado-Rodriguez SE, Vance RE, Hyde JL, Savan R, Mitchell PS, Daugherty MD. Host-specific sensing of coronaviruses and picornaviruses by the CARD8 inflammasome. PLoS Biol. 2023 Jun 8;21(6):e3002144. doi: 10.1371/journal.pbio.3002144. PMID: 37289745.
31. Andrews DDT, Vlok M, Akbari Bani D, Hay BN, Mohamud Y, Foster LJ, Luo H, Overall CM, Jan E. Cleavage of 14-3-3ε by the enteroviral 3C protease dampens RIG-I-mediated antiviral signaling. J Virol. 2023 Aug 31;97(8):e0060423. doi: 10.1128/jvi.00604-23. Epub 2023 Aug 9. PMID: 37555661.
